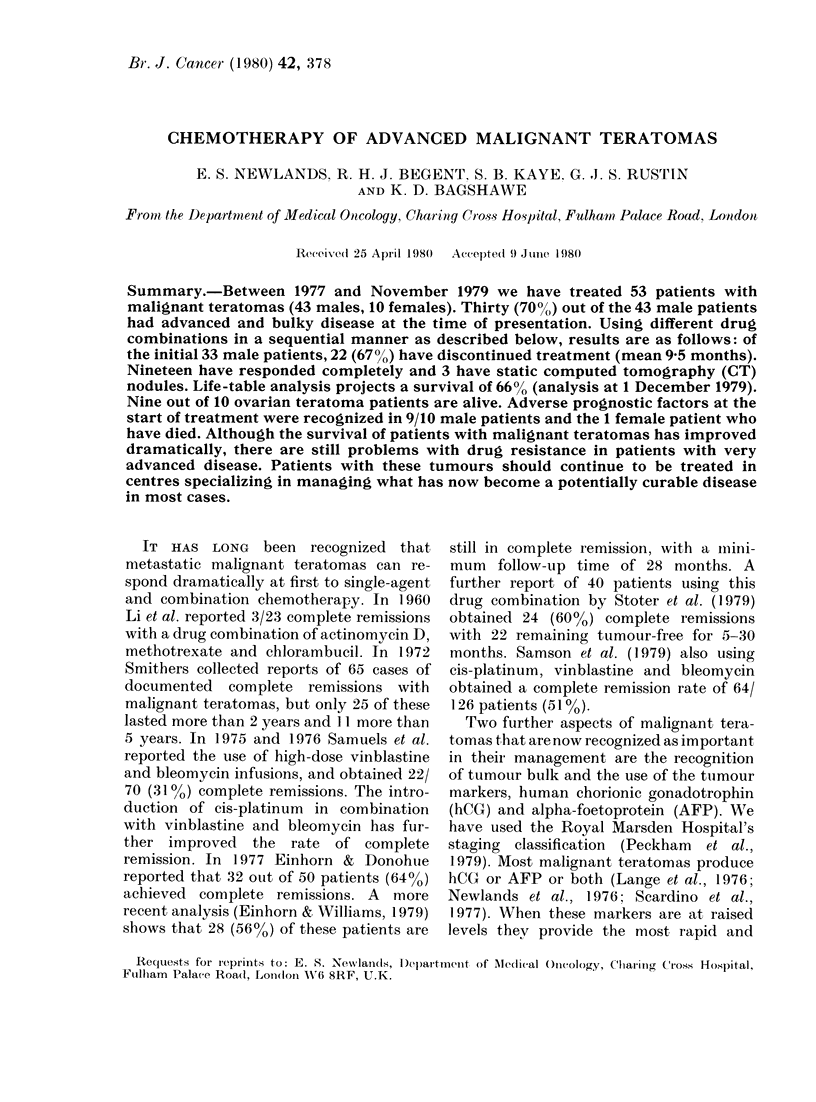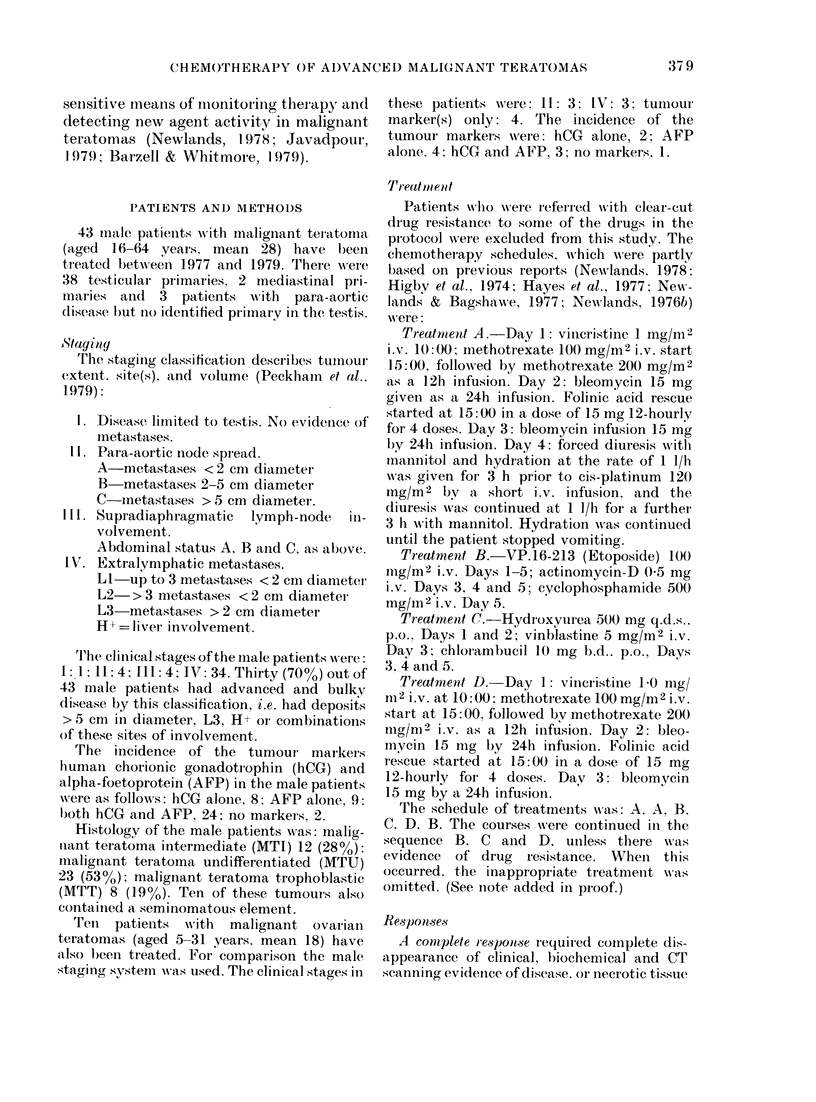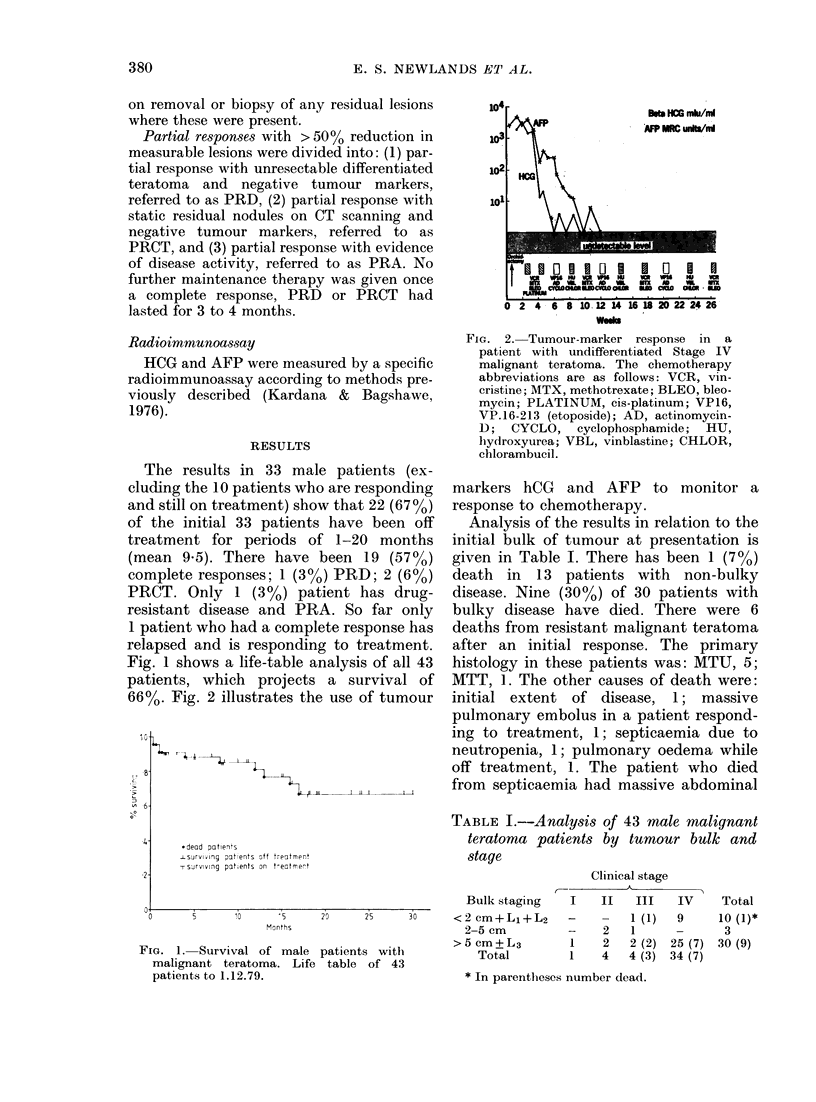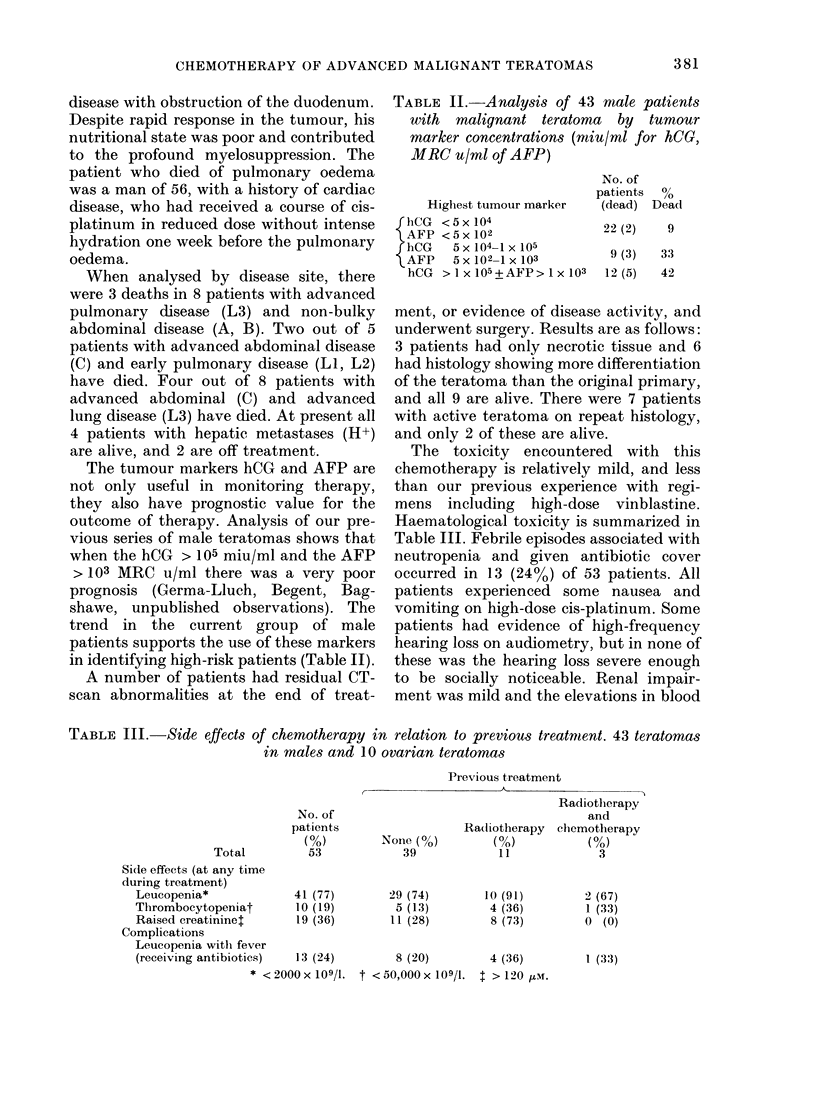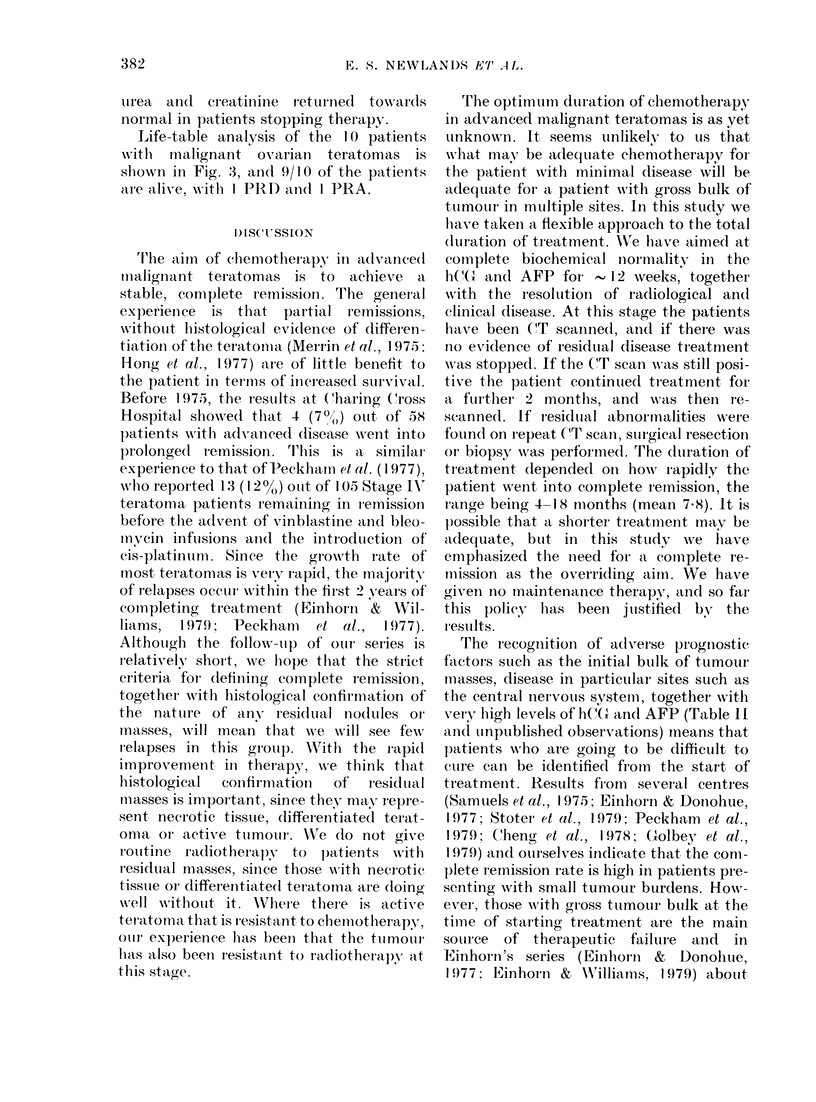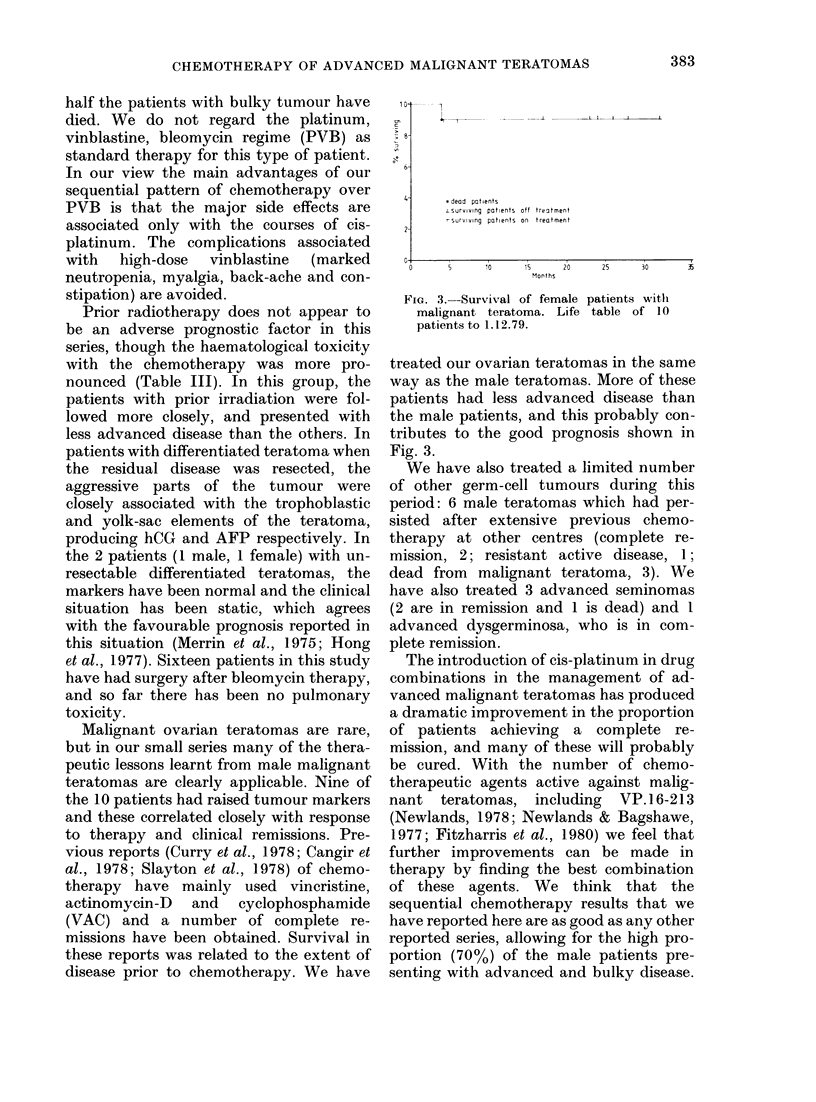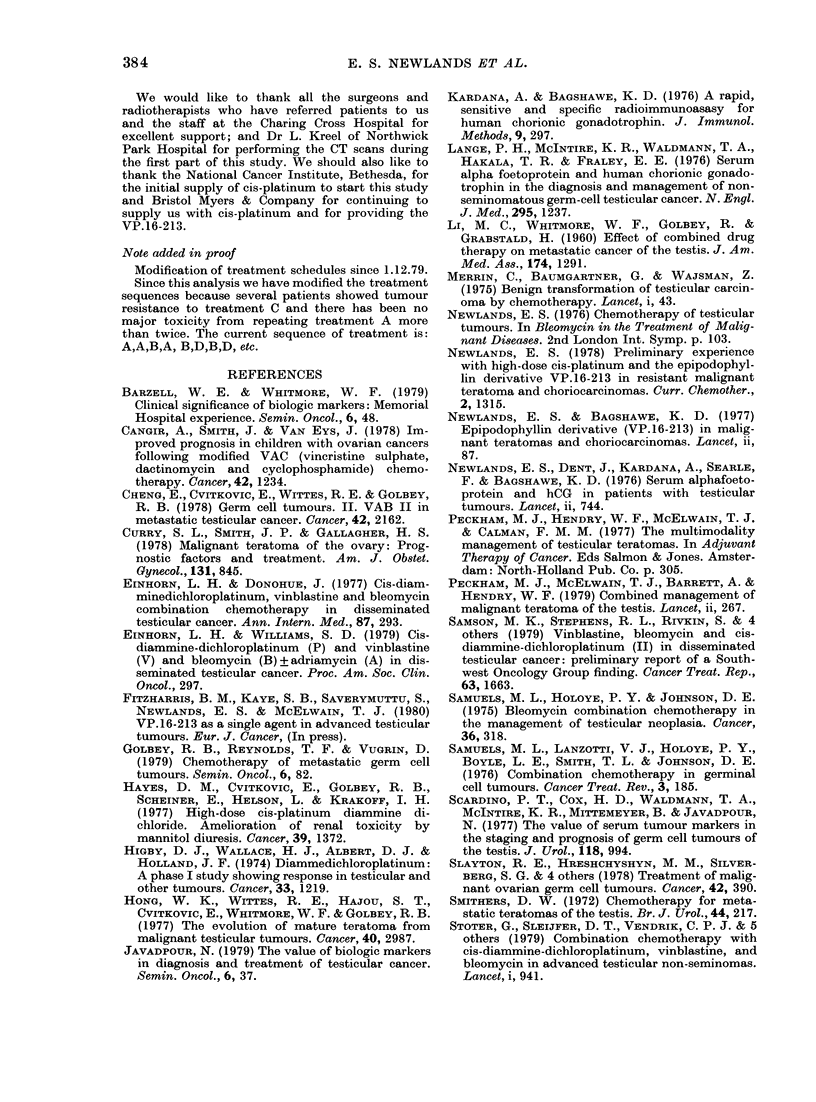# Chemotherapy of advanced malignant teratomas.

**DOI:** 10.1038/bjc.1980.248

**Published:** 1980-09

**Authors:** E. S. Newlands, R. H. Begent, S. B. Kaye, G. J. Rustin, K. D. Bagshawe

## Abstract

Between 1977 and November 1979 we have treated 53 patients with malignant teratomas (43 males, 10 females). Thirty (70%) out of the 43 male patients had advanced and bulky disease at the time of presentation. Using different drug combinations in a sequential manner as described below, results are as follows: of the initial 33 male patients, 22 (67%) have discontinued treatment (mean 9.5 months). Nineteen have responded completely and 3 have static computed tomography (CT) nodules. Life-table analysis projects a survival of 66% (analysis at 1 December 1979). Nine out of 10 ovarian teratoma patients are alive. Adverse prognostic factors at the start of treatment were recognized in 9/10 male patients and the 1 female patient who have died. Although the survival of patients with malignant teratomas has improved dramatically, there are still problems with drug resistance in patients with very advanced disease. Patients with these tumours should continue to be treated in centres specializing in managing what has now become a potentially curable disease in most cases.


*Br. J. Cancer* (1980) **42**, 378

### CHEMOTHERAPY OF ADVANCED MALIGNANT TERATOMAS

E. S. NEWLANDS, R. H. J. BEGENT, S. B. KAYE, G. J. S. RUSTIN AND K. D. BAGSHAWE

*From the Department of Medical Oncology, Charing Cross Hospital, Fulham Palace Road, London*

Received 25 April 1980 Accepted 9 June 1980

**Summary.—Between 1977 and November 1979 we have treated 53 patients with malignant teratomas (43 males, 10 females). Thirty (70%) out of the 43 male patients had advanced and bulky disease at the time of presentation. Using different drug combinations in a sequential manner as described below, results are as follows: of the initial 33 male patients, 22 (67%) have discontinued treatment (mean 9·5 months). Nineteen have responded completely and 3 have static computed tomography (CT) nodules. Life-table analysis projects a survival of 66% (analysis at 1 December 1979). Nine out of 10 ovarian teratoma patients are alive. Adverse prognostic factors at the start of treatment were recognized in 9/10 male patients and the 1 female patient who have died. Although the survival of patients with malignant teratomas has improved dramatically, there are still problems with drug resistance in patients with very advanced disease. Patients with these tumours should continue to be treated in centres specializing in managing what has now become a potentially curable disease in most cases.**

It has long been recognized that metastatic malignant teratomas can respond dramatically at first to single-agent and combination chemotherapy. In 1960 Li *et al.* reported 3/23 complete remissions with a drug combination of actinomycin D, methotrexate and chlorambucil. In 1972 Smithers collected reports of 65 cases of documented complete remissions with malignant teratomas, but only 25 of these lasted more than 2 years and 11 more than 5 years. In 1975 and 1976 Samuels *et al.* reported the use of high-dose vinblastine and bleomycin infusions, and obtained 22/70 (31%) complete remissions. The introduction of cis-platinum in combination with vinblastine and bleomycin has further improved the rate of complete remission. In 1977 Einhorn & Donohue reported that 32 out of 50 patients (64%) achieved complete remissions. A more recent analysis (Einhorn & Williams, 1979) shows that 28 (56%) of these patients are still in complete remission, with a minimum follow-up time of 28 months. A further report of 40 patients using this drug combination by Stoter *et al.* (1979) obtained 24 (60%) complete remissions with 22 remaining tumour-free for 5–30 months. Samson *et al.* (1979) also using cis-platinum, vinblastine and bleomycin obtained a complete remission rate of 64/126 patients (51%).

Two further aspects of malignant teratomas that are now recognized as important in their management are the recognition of tumour bulk and the use of the tumour markers, human chorionic gonadotrophin (hCG) and alpha-foetoprotein (AFP). We have used the Royal Marsden Hospital's staging classification (Peckham *et al.*, 1979). Most malignant teratomas produce hCG or AFP or both (Lange *et al.*, 1976; Newlands *et al.*, 1976; Scardino *et al.*, 1977). When these markers are at raised levels they provide the most rapid and

Requests for reprints to: E. S. Newlands, Department of Medical Oncology, Charing Cross Hospital, Fulham Palace Road, London W6 8RF, U.K.

sensitive means of monitoring therapy and detecting new agent activity in malignant teratomas (Newlands, 1978; Javadpour, 1979; Barzell & Whitmore, 1979).

### PATIENTS AND METHODS

43 male patients with malignant teratoma (aged 16–64 years, mean 28) have been treated between 1977 and 1979. There were 38 testicular primaries, 2 mediastinal primaries and 3 patients with para-aortic disease but no identified primary in the testis.

### *Staging*

The staging classification describes tumour extent, site(s), and volume (Peckham *et al.*, 1979):

I. Disease limited to testis. No evidence of metastases.
II. Para-aortic node spread.
A—metastases $<2$ cm diameter
B—metastases 2–5 cm diameter
C—metastases $>5$ cm diameter.
III. Supradiaphragmatic lymph-node involvement.
Abdominal status A, B and C, as above.
IV. Extralymphatic metastases.
L1—up to 3 metastases $<2$ cm diameter
L2—$>3$ metastases $<2$ cm diameter
L3—metastases $>2$ cm diameter
$H^+$ = liver involvement.

The clinical stages of the male patients were: I: 1; II: 4; III: 4; IV: 34. Thirty (70%) out of 43 male patients had advanced and bulky disease by this classification, *i.e.* had deposits $>5$ cm in diameter, L3, $H^+$ or combinations of these sites of involvement.

The incidence of the tumour markers human chorionic gonadotrophin (hCG) and alpha-foetoprotein (AFP) in the male patients were as follows: hCG alone, 8; AFP alone, 9; both hCG and AFP, 24; no markers, 2.

Histology of the male patients was: malignant teratoma intermediate (MTI) 12 (28%); malignant teratoma undifferentiated (MTU) 23 (53%); malignant teratoma trophoblastic (MTT) 8 (19%). Ten of these tumours also contained a seminomatous element.

Ten patients with malignant ovarian teratomas (aged 5–31 years, mean 18) have also been treated. For comparison the male staging system was used. The clinical stages in these patients were: II: 3; IV: 3; tumour marker(s) only: 4. The incidence of the tumour markers were: hCG alone, 2; AFP alone, 4; hCG and AFP, 3; no markers, 1.

### *Treatment*

Patients who were referred with clear-cut drug resistance to some of the drugs in the protocol were excluded from this study. The chemotherapy schedules, which were partly based on previous reports (Newlands, 1978; Higby *et al.*, 1974; Hayes *et al.*, 1977; Newlands & Bagshawe, 1977; Newlands, 1976*b*) were:

*Treatment A.*—Day 1: vincristine 1 mg/m$^2$ i.v. 10:00; methotrexate 100 mg/m$^2$ i.v. start 15:00, followed by methotrexate 200 mg/m$^2$ as a 12h infusion. Day 2: bleomycin 15 mg given as a 24h infusion. Folinic acid rescue started at 15:00 in a dose of 15 mg 12-hourly for 4 doses. Day 3: bleomycin infusion 15 mg by 24h infusion. Day 4: forced diuresis with mannitol and hydration at the rate of 1 l/h was given for 3 h prior to cis-platinum 120 mg/m$^2$ by a short i.v. infusion, and the diuresis was continued at 1 l/h for a further 3 h with mannitol. Hydration was continued until the patient stopped vomiting.

*Treatment B.*—VP.16-213 (Etoposide) 100 mg/m$^2$ i.v. Days 1–5; actinomycin-D 0·5 mg i.v. Days 3, 4 and 5; cyclophosphamide 500 mg/m$^2$ i.v. Day 5.

*Treatment C.*—Hydroxyurea 500 mg q.d.s., p.o., Days 1 and 2; vinblastine 5 mg/m$^2$ i.v. Day 3; chlorambucil 10 mg b.d., p.o., Days 3, 4 and 5.

*Treatment D.*—Day 1: vincristine 1·0 mg/m$^2$ i.v. at 10:00; methotrexate 100 mg/m$^2$ i.v. start at 15:00, followed by methotrexate 200 mg/m$^2$ i.v. as a 12h infusion. Day 2: bleomycin 15 mg by 24h infusion. Folinic acid rescue started at 15:00 in a dose of 15 mg 12-hourly for 4 doses. Day 3: bleomycin 15 mg by a 24h infusion.

The schedule of treatments was: A, A, B, C, D, B. The courses were continued in the sequence B, C and D, unless there was evidence of drug resistance. When this occurred, the inappropriate treatment was omitted. (See note added in proof.)

### *Responses*

*A complete response* required complete disappearance of clinical, biochemical and CT scanning evidence of disease, or necrotic tissue on removal or biopsy of any residual lesions where these were present.

*Partial responses* with >50% reduction in measurable lesions were divided into: (1) partial response with unresectable differentiated teratoma and negative tumour markers, referred to as PRD, (2) partial response with static residual nodules on CT scanning and negative tumour markers, referred to as PRCT, and (3) partial response with evidence of disease activity, referred to as PRA. No further maintenance therapy was given once a complete response, PRD or PRCT had lasted for 3 to 4 months.

### *Radioimmunoassay*

HCG and AFP were measured by a specific radioimmunoassay according to methods previously described (Kardana & Bagshawe, 1976).

### RESULTS

The results in 33 male patients (excluding the 10 patients who are responding and still on treatment) show that 22 (67%) of the initial 33 patients have been off treatment for periods of 1–20 months (mean 9·5). There have been 19 (57%) complete responses; 1 (3%) PRD; 2 (6%) PRCT. Only 1 (3%) patient has drug-resistant disease and PRA. So far only 1 patient who had a complete response has relapsed and is responding to treatment. Fig. 1 shows a life-table analysis of all 43 patients, which projects a survival of 66%. Fig. 2 illustrates the use of tumour markers hCG and AFP to monitor a response to chemotherapy.

FIG. 1.—Survival of male patients with malignant teratoma. Life table of 43 patients to 1.12.79.

FIG. 2.—Tumour-marker response in a patient with undifferentiated Stage IV malignant teratoma. The chemotherapy abbreviations are as follows: VCR, vincristine; MTX, methotrexate; BLEO, bleomycin; PLATINUM, cis-platinum; VP16, VP.16-213 (etoposide); AD, actinomycin-D; CYCLO, cyclophosphamide; HU, hydroxyurea; VBL, vinblastine; CHLOR, chlorambucil.

Analysis of the results in relation to the initial bulk of tumour at presentation is given in Table I. There has been 1 (7%) death in 13 patients with non-bulky disease. Nine (30%) of 30 patients with bulky disease have died. There were 6 deaths from resistant malignant teratoma after an initial response. The primary histology in these patients was: MTU, 5; MTT, 1. The other causes of death were: initial extent of disease, 1; massive pulmonary embolus in a patient responding to treatment, 1; septicaemia due to neutropenia, 1; pulmonary oedema while off treatment, 1. The patient who died from septicaemia had massive abdominal

TABLE I.—*Analysis of 43 male malignant teratoma patients by tumour bulk and stage*

| | Clinical stage | | | | |
|---|---|---|---|---|---|
| Bulk staging | I | II | III | IV | Total |
| $<2$ cm $+L_1+L_2$ | – | – | 1 (1) | 9 | 10 (1)* |
| 2–5 cm | – | 2 | 1 | – | 3 |
| $>5$ cm $\pm L_3$ | 1 | 2 | 2 (2) | 25 (7) | 30 (9) |
| Total | 1 | 4 | 4 (3) | 34 (7) | |

* In parentheses number dead.

disease with obstruction of the duodenum. Despite rapid response in the tumour, his nutritional state was poor and contributed to the profound myelosuppression. The patient who died of pulmonary oedema was a man of 56, with a history of cardiac disease, who had received a course of cis-platinum in reduced dose without intense hydration one week before the pulmonary oedema.

When analysed by disease site, there were 3 deaths in 8 patients with advanced pulmonary disease (L3) and non-bulky abdominal disease (A, B). Two out of 5 patients with advanced abdominal disease (C) and early pulmonary disease (L1, L2) have died. Four out of 8 patients with advanced abdominal (C) and advanced lung disease (L3) have died. At present all 4 patients with hepatic metastases ($H^+$) are alive, and 2 are off treatment.

The tumour markers hCG and AFP are not only useful in monitoring therapy, they also have prognostic value for the outcome of therapy. Analysis of our previous series of male teratomas shows that when the hCG $> 10^5$ miu/ml and the AFP $> 10^3$ MRC u/ml there was a very poor prognosis (Germa-Lluch, Begent, Bagshawe, unpublished observations). The trend in the current group of male patients supports the use of these markers in identifying high-risk patients (Table II).

A number of patients had residual CT-scan abnormalities at the end of treatment, or evidence of disease activity, and underwent surgery. Results are as follows: 3 patients had only necrotic tissue and 6 had histology showing more differentiation of the teratoma than the original primary, and all 9 are alive. There were 7 patients with active teratoma on repeat histology, and only 2 of these are alive.

The toxicity encountered with this chemotherapy is relatively mild, and less than our previous experience with regimens including high-dose vinblastine. Haematological toxicity is summarized in Table III. Febrile episodes associated with neutropenia and given antibiotic cover occurred in 13 (24%) of 53 patients. All patients experienced some nausea and vomiting on high-dose cis-platinum. Some patients had evidence of high-frequency hearing loss on audiometry, but in none of these was the hearing loss severe enough to be socially noticeable. Renal impairment was mild and the elevations in blood

TABLE II.—*Analysis of* 43 *male patients with malignant teratoma by tumour marker concentrations* (*miu/ml for hCG, MRC u/ml of AFP*)

| Highest tumour marker | No. of patients (dead) | % Dead |
|---|---|---|
| hCG $< 5 \times 10^4$; AFP $< 5 \times 10^2$ | 22 (2) | 9 |
| hCG $5 \times 10^4$–$1 \times 10^5$; AFP $5 \times 10^2$–$1 \times 10^3$ | 9 (3) | 33 |
| hCG $> 1 \times 10^5 \pm$ AFP $> 1 \times 10^3$ | 12 (5) | 42 |

TABLE III.—*Side effects of chemotherapy in relation to previous treatment.* 43 *teratomas in males and* 10 *ovarian teratomas*

| | No. of patients (%) | Previous treatment: None (%) | Radiotherapy (%) | Radiotherapy and chemotherapy (%) |
|---|---|---|---|---|
| Total | 53 | 39 | 11 | 3 |
| Side effects (at any time during treatment) | | | | |
| Leucopenia* | 41 (77) | 29 (74) | 10 (91) | 2 (67) |
| Thrombocytopenia† | 10 (19) | 5 (13) | 4 (36) | 1 (33) |
| Raised creatinine‡ | 19 (36) | 11 (28) | 8 (73) | 0 (0) |
| Complications | | | | |
| Leucopenia with fever (receiving antibiotics) | 13 (24) | 8 (20) | 4 (36) | 1 (33) |

urea and creatinine returned towards normal in patients stopping therapy.

Life-table analysis of the 10 patients with malignant ovarian teratomas is shown in Fig. 3, and 9/10 of the patients are alive, with 1 PRD and 1 PRA.

### DISCUSSION

The aim of chemotherapy in advanced malignant teratomas is to achieve a stable, complete remission. The general experience is that partial remissions, without histological evidence of differentiation of the teratoma (Merrin *et al.*, 1975; Hong *et al.*, 1977) are of little benefit to the patient in terms of increased survival. Before 1975, the results at Charing Cross Hospital showed that 4 (7%) out of 58 patients with advanced disease went into prolonged remission. This is a similar experience to that of Peckham *et al.* (1977), who reported 13 (12%) out of 105 Stage IV teratoma patients remaining in remission before the advent of vinblastine and bleomycin infusions and the introduction of cis-platinum. Since the growth rate of most teratomas is very rapid, the majority of relapses occur within the first 2 years of completing treatment (Einhorn & Williams, 1979; Peckham *et al.*, 1977). Although the follow-up of our series is relatively short, we hope that the strict criteria for defining complete remission, together with histological confirmation of the nature of any residual nodules or masses, will mean that we will see few relapses in this group. With the rapid improvement in therapy, we think that histological confirmation of residual masses is important, since they may represent necrotic tissue, differentiated teratoma or active tumour. We do not give routine radiotherapy to patients with residual masses, since those with necrotic tissue or differentiated teratoma are doing well without it. Where there is active teratoma that is resistant to chemotherapy, our experience has been that the tumour has also been resistant to radiotherapy at this stage.

The optimum duration of chemotherapy in advanced malignant teratomas is as yet unknown. It seems unlikely to us that what may be adequate chemotherapy for the patient with minimal disease will be adequate for a patient with gross bulk of tumour in multiple sites. In this study we have taken a flexible approach to the total duration of treatment. We have aimed at complete biochemical normality in the hCG and AFP for ~12 weeks, together with the resolution of radiological and clinical disease. At this stage the patients have been CT scanned, and if there was no evidence of residual disease treatment was stopped. If the CT scan was still positive the patient continued treatment for a further 2 months, and was then rescanned. If residual abnormalities were found on repeat CT scan, surgical resection or biopsy was performed. The duration of treatment depended on how rapidly the patient went into complete remission, the range being 4–18 months (mean 7·8). It is possible that a shorter treatment may be adequate, but in this study we have emphasized the need for a complete remission as the overriding aim. We have given no maintenance therapy, and so far this policy has been justified by the results.

The recognition of adverse prognostic factors such as the initial bulk of tumour masses, disease in particular sites such as the central nervous system, together with very high levels of hCG and AFP (Table II and unpublished observations) means that patients who are going to be difficult to cure can be identified from the start of treatment. Results from several centres (Samuels *et al.*, 1975; Einhorn & Donohue, 1977; Stoter *et al.*, 1979; Peckham *et al.*, 1979; Cheng *et al.*, 1978; Golbey *et al.*, 1979) and ourselves indicate that the complete remission rate is high in patients presenting with small tumour burdens. However, those with gross tumour bulk at the time of starting treatment are the main source of therapeutic failure and in Einhorn's series (Einhorn & Donohue, 1977; Einhorn & Williams, 1979) about half the patients with bulky tumour have died. We do not regard the platinum, vinblastine, bleomycin regime (PVB) as standard therapy for this type of patient. In our view the main advantages of our sequential pattern of chemotherapy over PVB is that the major side effects are associated only with the courses of cis-platinum. The complications associated with high-dose vinblastine (marked neutropenia, myalgia, back-ache and constipation) are avoided.

Prior radiotherapy does not appear to be an adverse prognostic factor in this series, though the haematological toxicity with the chemotherapy was more pronounced (Table III). In this group, the patients with prior irradiation were followed more closely, and presented with less advanced disease than the others. In patients with differentiated teratoma when the residual disease was resected, the aggressive parts of the tumour were closely associated with the trophoblastic and yolk-sac elements of the teratoma, producing hCG and AFP respectively. In the 2 patients (1 male, 1 female) with unresectable differentiated teratomas, the markers have been normal and the clinical situation has been static, which agrees with the favourable prognosis reported in this situation (Merrin *et al.*, 1975; Hong *et al.*, 1977). Sixteen patients in this study have had surgery after bleomycin therapy, and so far there has been no pulmonary toxicity.

Malignant ovarian teratomas are rare, but in our small series many of the therapeutic lessons learnt from male malignant teratomas are clearly applicable. Nine of the 10 patients had raised tumour markers and these correlated closely with response to therapy and clinical remissions. Previous reports (Curry *et al.*, 1978; Cangir *et al.*, 1978; Slayton *et al.*, 1978) of chemotherapy have mainly used vincristine, actinomycin-D and cyclophosphamide (VAC) and a number of complete remissions have been obtained. Survival in these reports was related to the extent of disease prior to chemotherapy. We have

Fig. 3.—Survival of female patients with malignant teratoma. Life table of 10 patients to 1.12.79.

treated our ovarian teratomas in the same way as the male teratomas. More of these patients had less advanced disease than the male patients, and this probably contributes to the good prognosis shown in Fig. 3.

We have also treated a limited number of other germ-cell tumours during this period: 6 male teratomas which had persisted after extensive previous chemotherapy at other centres (complete remission, 2; resistant active disease, 1; dead from malignant teratoma, 3). We have also treated 3 advanced seminomas (2 are in remission and 1 is dead) and 1 advanced dysgerminosa, who is in complete remission.

The introduction of cis-platinum in drug combinations in the management of advanced malignant teratomas has produced a dramatic improvement in the proportion of patients achieving a complete remission, and many of these will probably be cured. With the number of chemotherapeutic agents active against malignant teratomas, including VP.16-213 (Newlands, 1978; Newlands & Bagshawe, 1977; Fitzharris *et al.*, 1980) we feel that further improvements can be made in therapy by finding the best combination of these agents. We think that the sequential chemotherapy results that we have reported here are as good as any other reported series, allowing for the high proportion (70%) of the male patients presenting with advanced and bulky disease.

We would like to thank all the surgeons and radiotherapists who have referred patients to us and the staff at the Charing Cross Hospital for excellent support; and Dr L. Kreel of Northwick Park Hospital for performing the CT scans during the first part of this study. We should also like to thank the National Cancer Institute, Bethesda, for the initial supply of cis-platinum to start this study and Bristol Myers & Company for continuing to supply us with cis-platinum and for providing the VP.16-213.

*Note added in proof*

Modification of treatment schedules since 1.12.79. Since this analysis we have modified the treatment sequences because several patients showed tumour resistance to treatment C and there has been no major toxicity from repeating treatment A more than twice. The current sequence of treatment is: A,A,B,A, B,D,B,D, *etc.*

### REFERENCES

BARZELL, W. E. & WHITMORE, W. F. (1979) Clinical significance of biologic markers: Memorial Hospital experience. *Semin. Oncol.*, **6**, 48.

CANGIR, A., SMITH, J. & VAN EYS, J. (1978) Improved prognosis in children with ovarian cancers following modified VAC (vincristine sulphate, dactinomycin and cyclophosphamide) chemotherapy. *Cancer*, **42**, 1234.

CHENG, E., CVITKOVIC, E., WITTES, R. E. & GOLBEY, R. B. (1978) Germ cell tumours. II. VAB II in metastatic testicular cancer. *Cancer*, **42**, 2162.

CURRY, S. L., SMITH, J. P. & GALLAGHER, H. S. (1978) Malignant teratoma of the ovary: Prognostic factors and treatment. *Am. J. Obstet. Gynecol.*, **131**, 845.

EINHORN, L. H. & DONOHUE, J. (1977) Cis-diamminedichloroplatinum, vinblastine and bleomycin combination chemotherapy in disseminated testicular cancer. *Ann. Intern. Med.*, **87**, 293.

EINHORN, L. H. & WILLIAMS, S. D. (1979) Cis-diammine-dichloroplatinum (P) and vinblastine (V) and bleomycin (B) ± adriamycin (A) in disseminated testicular cancer. *Proc. Am. Soc. Clin. Oncol.*, 297.

FITZHARRIS, B. M., KAYE, S. B., SAVERYMUTTU, S., NEWLANDS, E. S. & MCELWAIN, T. J. (1980) VP.16-213 as a single agent in advanced testicular tumours. *Eur. J. Cancer*, (In press).

GOLBEY, R. B., REYNOLDS, T. F. & VUGRIN, D. (1979) Chemotherapy of metastatic germ cell tumours. *Semin. Oncol.*, **6**, 82.

HAYES, D. M., CVITKOVIC, E., GOLBEY, R. B., SCHEINER, E., HELSON, L. & KRAKOFF, I. H. (1977) High-dose cis-platinum diammine dichloride. Amelioration of renal toxicity by mannitol diuresis. *Cancer*, **39**, 1372.

HIGBY, D. J., WALLACE, H. J., ALBERT, D. J. & HOLLAND, J. F. (1974) Diammedichloroplatinum: A phase I study showing response in testicular and other tumours. *Cancer*, **33**, 1219.

HONG, W. K., WITTES, R. E., HAJOU, S. T., CVITKOVIC, E., WHITMORE, W. F. & GOLBEY, R. B. (1977) The evolution of mature teratoma from malignant testicular tumours. *Cancer*, **40**, 2987.

JAVADPOUR, N. (1979) The value of biologic markers in diagnosis and treatment of testicular cancer. *Semin. Oncol.*, **6**, 37.

KARDANA, A. & BAGSHAWE, K. D. (1976) A rapid, sensitive and specific radioimmunoasasy for human chorionic gonadotrophin. *J. Immunol. Methods*, **9**, 297.

LANGE, P. H., MCINTIRE, K. R., WALDMANN, T. A., HAKALA, T. R. & FRALEY, E. E. (1976) Serum alpha foetoprotein and human chorionic gonadotrophin in the diagnosis and management of non-seminomatous germ-cell testicular cancer. *N. Engl. J. Med.*, **295**, 1237.

LI, M. C., WHITMORE, W. F., GOLBEY, R. & GRABSTALD, H. (1960) Effect of combined drug therapy on metastatic cancer of the testis. *J. Am. Med. Ass.*, **174**, 1291.

MERRIN, C., BAUMGARTNER, G. & WAJSMAN, Z. (1975) Benign transformation of testicular carcinoma by chemotherapy. *Lancet*, i, 43.

NEWLANDS, E. S. (1976) Chemotherapy of testicular tumours. In *Bleomycin in the Treatment of Malignant Diseases*. 2nd London Int. Symp. p. 103.

NEWLANDS, E. S. (1978) Preliminary experience with high-dose cis-platinum and the epipodophyllin derivative VP.16-213 in resistant malignant teratoma and choriocarcinomas. *Curr. Chemother.*, **2**, 1315.

NEWLANDS, E. S. & BAGSHAWE, K. D. (1977) Epipodophyllin derivative (VP.16-213) in malignant teratomas and choriocarcinomas. *Lancet*, ii, 87.

NEWLANDS, E. S., DENT, J., KARDANA, A., SEARLE, F. & BAGSHAWE, K. D. (1976) Serum alphafoetoprotein and hCG in patients with testicular tumours. *Lancet*, ii, 744.

PECKHAM, M. J., HENDRY, W. F., MCELWAIN, T. J. & CALMAN, F. M. M. (1977) The multimodality management of testicular teratomas. In *Adjuvant Therapy of Cancer*. Eds Salmon & Jones. Amsterdam: North-Holland Pub. Co. p. 305.

PECKHAM, M. J., MCELWAIN, T. J., BARRETT, A. & HENDRY, W. F. (1979) Combined management of malignant teratoma of the testis. *Lancet*, ii, 267.

SAMSON, M. K., STEPHENS, R. L., RIVKIN, S. & 4 others (1979) Vinblastine, bleomycin and cis-diammine-dichloroplatinum (II) in disseminated testicular cancer: preliminary report of a Southwest Oncology Group finding. *Cancer Treat. Rep.*, **63**, 1663.

SAMUELS, M. L., HOLOYE, P. Y. & JOHNSON, D. E. (1975) Bleomycin combination chemotherapy in the management of testicular neoplasia. *Cancer*, **36**, 318.

SAMUELS, M. L., LANZOTTI, V. J., HOLOYE, P. Y., BOYLE, L. E., SMITH, T. L. & JOHNSON, D. E. (1976) Combination chemotherapy in germinal cell tumours. *Cancer Treat. Rev.*, **3**, 185.

SCARDINO, P. T., COX, H. D., WALDMANN, T. A., MCINTIRE, K. R., MITTEMEYER, B. & JAVADPOUR, N. (1977) The value of serum tumour markers in the staging and prognosis of germ cell tumours of the testis. *J. Urol.*, **118**, 994.

SLAYTON, R. E., HRESHCHYSHYN, M. M., SILVERBERG, S. G. & 4 others (1978) Treatment of malignant ovarian germ cell tumours. *Cancer*, **42**, 390.

SMITHERS, D. W. (1972) Chemotherapy for metastatic teratomas of the testis. *Br. J. Urol.*, **44**, 217.

STOTER, G., SLEIJFER, D. T., VENDRIK, C. P. J. & 5 others (1979) Combination chemotherapy with cis-diammine-dichloroplatinum, vinblastine, and bleomycin in advanced testicular non-seminomas. *Lancet*, i, 941.